# Superhydrophobic, Magnetic Aerogels Based on Nanocellulose Fibers Derived from Harakeke for Oily Wastewater Remediation

**DOI:** 10.3390/polym15193941

**Published:** 2023-09-29

**Authors:** Yitong Zhai, Xiaowen Yuan

**Affiliations:** Future Fibers Laboratory, School of Engineering, Computer and Mathematical Sciences, Auckland University of Technology, Auckland 1010, New Zealand; yitong.zhai@autuni.ac.nz

**Keywords:** aerogel, cellulose, harakeke, magnetic, oil adsorption, reusability

## Abstract

Cellulose-based aerogels have been seen as a promising sorbent for oil and organic pollutant cleaning; however, their intrinsic hydrophilicity and difficulty of recycling has hindered their practical application. In this work, a superhydrophobic, magnetic cellulose-based aerogel was fabricated as a highly efficient sorbent for the adsorption of oils and organic solvents. The aerogel was prepared via a simple freeze-drying method, followed by chemical vapor deposition (CVD). The incorporation of Fe_3_O_4_ nanoparticles into the aerogel not only makes it responsive to external magnetic field, but also contributes to the better hydrophobicity of the aerogel, in which the water contact angle (WCA) was about 20° higher than the aerogel without loading with Fe_3_O_4_ nanoparticles. The adsorption test showed that the resultant aerogel can selectively adsorb a wide range of oils and organic solvents from oil/water mixtures with a high adsorption capacity (up to 113.49 g/g for silicone oil). It can retain about 50% of its adsorption capacity even after 10 adsorption–squeezing cycles, which indicates its outstanding reusability. Moreover, the aerogels can be easily controlled by an external magnet, which is preferred for the adsorption of oily contaminants in harsh environments and enhanced the recyclability of the aerogel. We believe that this study provides a green and convenient approach for the practical fabrication of cellulose-based oil sorbents.

## 1. Introduction

It is widely recognized that there is an urgent need to address the issue of water pollution caused by petroleum and organic solvents [1]. Various methods, such as in situ burning [2], flotation [3], centrifugation [4], and bioremediation [5] have been applied to solve this problem. However, those methods show many disadvantages, such as secondary pollution, high-cost, and high-energy consumption. Owing to the high efficiency and ease of operation, physical adsorption has been considered to be a preferable approach to other methods. Traditionally used synthetic polymer-based sorbents, including polyurethane foam, melamine foam, and polypropylene, have been modified through various methods, including particle deposition and polymerization, to alter their surface wettability to achieve the selective adsorption of oil [6,7]. However, those synthetic polymer-based oil sorbents not only show low-efficiency and non-biodegradability, but also suffer from complicated preparation processes and non-satisfactory adsorption capacities [8]. Nowadays, the concept of sustainable development has attracted much attention from researchers. The demand for renewable carbohydrate polymers to replace petroleum-based polymers has continuously grown [9,10]. As a renewable, biocompatible, biodegradable, and most abundant natural polymer, cellulose can be extracted from plants, animals, tunicates, algae, and some bacteria. Owing to its abundancy and low cost, cellulose is now receiving widespread attention from researchers as a promising alternative to fossil fuel-based polymers [11].

Since first being reported by Kistler in 1931 [12], aerogels have developed a broad variety of different types, ranging from the first generation of silica-based to the third generation of cellulose-based types [13]. Aerogels are ultralight 3D porous materials with many fascinating properties, such as low density, high porosity, and expansive specific surface area. Aerogels have been extensively studied for a wide range of applications from thermal insulation and drug delivery to the provision of catalyst carriers [14]. Furthermore, the high volume-to-weight ratios of aerogels make them ideal for sorbent applications [8]. Different types of aerogels have been investigated and have shown promising results for oil sorption, such as synthetic silicone [15], synthetic polymer-based [16], and graphene-based aerogels [17]. However, the silica aerogels suffer from poor mechanical strength and fragility [18], and synthetic polymer-based aerogels are not biodegradable and their accumulation in the ecosystem makes the problem more serious [19]. The high cost and complicated fabrication process are the main drawbacks of graphene-based aerogels. Recently, considerable attention has been paid to developing novel cellulose-based aerogels for oil sorption and oil/water mixture separation. For example, Zhang et al. [20] modified microfibrillated cellulose with Vinyltrimethoxysilane (VTMO) and prepared hydrophobic aerogel via freeze-drying. The modified microfibrillated cellulose aerogel was used as an oil sorbent for oil/water mixture separation and exhibited a high adsorption capacity up to 130.1 g/g. Chen et al. [21] immersed the cellulose aerogel into hydrolyzed silicone alcohols, in which the silanol reacted with the cellulose hydroxyl groups to prepare hydrophobic cellulose-based aerogels for oil sorption.

However, the difficulty of recycling oil-adsorbed aerogels hinders their practical application. Preparing magnetic and hydrophobic cellulose aerogel provides a promising solution to this issue. Herein, a facile and cost-effective method was developed to prepare Fe_3_O_4_ nanoparticle-loaded, low-density, high-porosity, superhydrophobic, and magnetic cellulose aerogel (HMCA) from harakeke (a New Zealand native plant). With the incorporation of Fe_3_O_4_ nanoparticles, the HMCA can be controlled to move by an external magnet, which greatly facilitates the recycling of oil saturated aerogels and the application of aerogels in harsh environments. The prepared HMCA exhibited outstanding adsorption and retention capacity towards a variety of oils and organic solvents and excellent oil adsorption selectivity in oil/water mixtures. The adsorption capacity of HMCA is the highest for silicone oil of 113.49 g/g. The aerogel also exhibited good reusability, where about 50% of its original adsorption capacity can be retained after 10 adsorption–squeezing cycles. Moreover, the aerogel only showed an about 10% reduction in the adsorption capacity if the adsorbed oils were removed via washing. Therefore, the HMCA prepared in this work has the potential to become an efficient and sustainable oil absorbent for treating oil pollution in water.

## 2. Materials and Methods

### 2.1. Materials

Pure cellulose fibers (a SEM image of the fibers is shown in Figure S1) were extracted from harakeke according to the method from our previous work [22]. Glutaraldehyde solution (25%, Alfa Aesar, Haverhill, MA, USA) was used. Fe_3_O_4_ nanoparticles, methyltrichlorosilane (MTCS), sulfuric acid (H_2_SO_4_, 1.0 vol%), and ethanol were obtained from Aladdin Chemistry Co. Ltd. (Shanghai, China). All chemicals were analytical grade, and distilled water was used throughout.

### 2.2. Synthesis of Pristine Cellulose Aerogel (CA)

In total, 0.5 g of the extracted cellulose was magnetically stirred in 99.5 g of distilled water at 25 °C for 48 h to allow the aerogels to completely swell. The mixture was then put into a homogenizer (SHW400R, Shanghai Weisheng Electrical Instrument Co., Ltd., Shanghai, China) at 10,000 rpm for 2 h, followed by ultrasonication treatment (UP400St, Hielscher, Teltow, Germany) at an output power of 600 W for 2 h in an ice/water bath so that a homogeneous cellulose microfibril suspension of 0.5 wt% was formed. Subsequently, 150 µL of glutaraldehyde (25 wt%) and 50 µL of sulfuric acid (2 vol%) were added dropwise into 30 g of CNF suspension (0.5 wt%) via magnetic stirring for 1 h, followed by heating in an oven at 75 °C for 3 h to promote crosslinking. Next, the solution was frozen at −196 °C using liquid nitrogen and freeze-dried (LGJ-12S, Songyuan Freeze Dryer, Songyuan, China) at −50 °C for 48 h to produce cellulose aerogel.

### 2.3. Preparation of Magnetic Fe_3_O_4_ Nanoparticle-Coated Cellulose Aerogels (MCA)

In order to attain the optimal addition of Fe_3_O_4_ nanoparticles for MCA, different amounts of Fe_3_O_4_ nanoparticles (0.2, 0.4, 0.6, and 0.8 wt%) were added to the glutaraldehyde and cellulose suspension to prepare MCAs with different Fe_3_O_4_ loadings. The Fe_3_O_4_/cellulose mixture suspension was magnetically stirred for 1 h at room temperature, followed by ultrasonication in the ice/water bath for another 20 min to remove any bubbles and uniformly disperse the Fe_3_O_4_ nanoparticles. The resultant bubble-free solution was then placed in a vacuum oven at 75 °C for 3 h to promote crosslinking. Subsequently, the crosslinked mixture was poured into a brass mold and frozen using liquid nitrogen at −196 °C and then freeze-dried for 48 h at −50 °C.

### 2.4. Preparation of Super-Hydrophobic Magnetic Cellulose Aerogels (HMCA)

The pristine MCA was hydrophilic because of the presence of large amounts of surface hydroxyl groups on the cellulose fiber. Therefore, hydrophobization was needed to modify the MCA, making it more suitable for oil adsorption. Silanization was carried out through chemical vapor deposition, using MTCS as the precursor. The obtained MCA was placed in a desiccator, and an open glass vial containing 5 mL of MTCS was placed next to the MCA in the same desiccator. Then, the desiccator was tightly sealed and heated in an oven at 50 °C for 12 h.

### 2.5. Characterization

The morphology of the aerogels was observed via scanning electron microscopy (SEM) (ZEISS Sigma 300, Carl Zeiss, Oberkochen, Germany) at a working voltage of 5 kV. The SEM was equipped with an energy-dispersive X-ray (EDX) spectrometer for elemental analysis. The chemical structure of the aerogels was characterized using a Fourier transform infrared spectrometer (FT-IR) (IRTracer-10; Shimadzu Co. Ltd., Tokyo, Japan) within 600–4000 cm^−1^, with a resolution of 4 cm^−1^, and X-ray diffraction (D8 Advance, Bruker, Billerica, MA, USA) operating with Cu-Kα radiation (λ = 1.5418 Å) at a scan rate of 4°/min at 40 kV/20 mA, ranging from 5 to 60°. The surface elemental analyses of the aerogels were characterized via X-ray photoelectron spectroscopy (XPS) with Al Ka X-ray radiation (Thermo Fisher, Waltham, MA, USA). The thermal stability of the sample was characterized via thermogravimetric analysis (TG) using a TG analyzer (Q600, STA, New Castle, DE, USA). The sample was heated in air at a heating rate of 10 °C min^−1^ from room temperature to 800 °C. The surface wettability of the aerogels was determined by measuring static water contact angle using a contact angle goniometer (OCA20, Dataphysics, Filderstadt, Germany) at ambient temperature. The magnetic measurement was performed using a vibrating sample magnetometer (VSM, LH-3, Nanjing NanDa Instrument Co., Ltd., Nanjing, China). Hysteresis loops were generated for the determination of the aerogel saturation magnetization (Ms) and coercivity (Hc).

The density of the aerogels was calculated via Equation (1), where the mass of the sample was measured using an electronic balance. Three parallel tests were carried out on each sample to find the average result [23].

(1) $$\rho =\frac{m}{v}$$

where *ρ* is the density of the aerogel, and *m* and *v* are the mass (g) and volume (cm^3^) of the sample, respectively.

The porosity of the sample was calculated via Equation (2).

(2) $$P\left(\%\right)=\frac{V-\left(\frac{m}{\rho_{c}}\right)}{V}\times 100\%$$

where *P* is the porosity of the aerogel, *m* and *v* are the mass (g) and volume (cm^3^) of the sample, and *ρ_c_* is the bulk density of the cellulose scaffold, which is the bulk density of cellulose (*ρ_c_* = 1.528 g/cm^3^) [24].

The adsorption capacity of HMCA was determined based on the method reported in a previous work. In brief, a measured weight of HMCA was immersed in different oils and solvents at room temperature for sufficient time to reach adsorption saturation. We removed the sample from the oil to drain the surface excess and weighed the sample again. The oil adsorption capacity of the HMCA was calculated based on Equation (3)

(3) $$K=\frac{W-W_{o}}{W_{o}}\times 100\%$$

where *K* is the adsorption capacity of HMCA, and *W_o_* and *W* are the weight before and after oil adsorption, respectively [25].

## 3. Results and Discussion

### 3.1. Fabrication and Structural Characterization of CA, MCA, and HMCA

**Figure 1:**
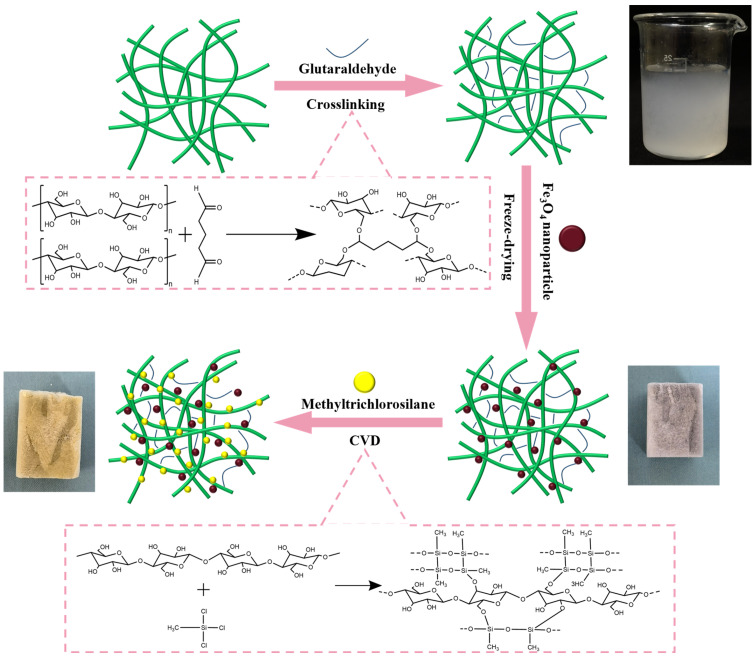
The schematic illustration of the preparation process of superhydrophobic magnetic cellulose aerogels.

**Figure 2:**
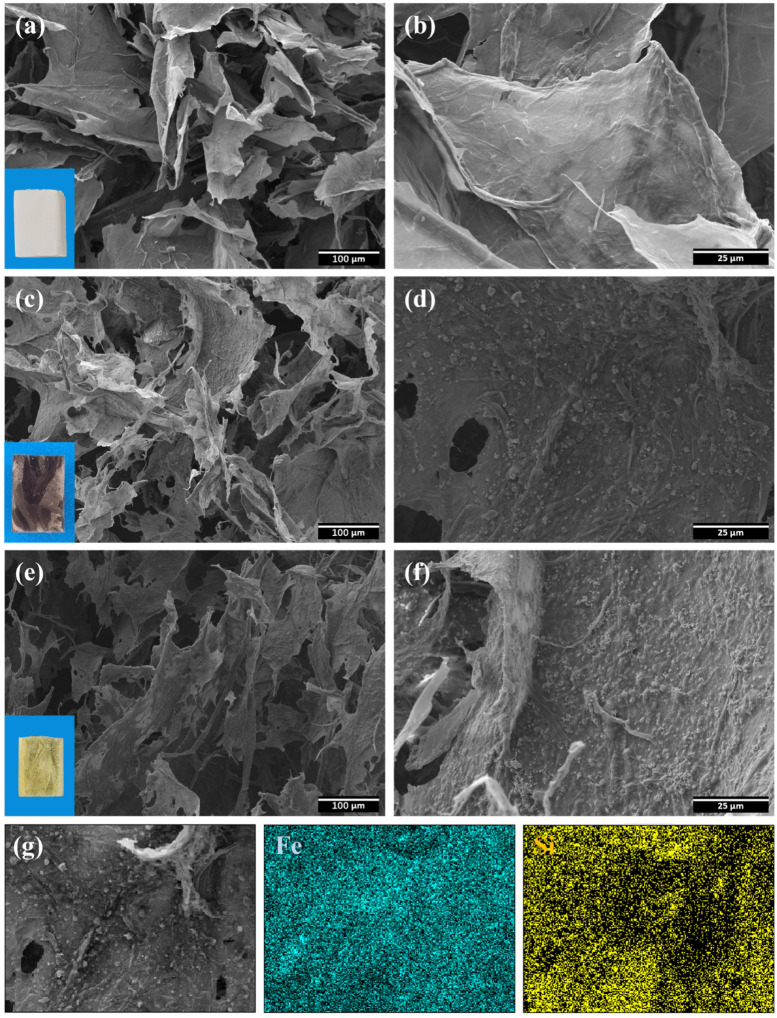
SEM images of (**a**,**b**) CA at different magnifications. (**c**,**d**) MCA at different magnifications, and (**e**,**f**) HMCA at different magnifications. The corresponding digital camera photos of each aerogel are at the bottom-left corner of (**a**,**c**,**e**). (**g**) EDX elemental mapping of HMCA, with blue and yellow corresponding to Fe and Si, respectively.

Figure 1 is a schematic illustration of the preparation process of HMCA, and the corresponding photographs are of each intermediate aerogel. The cellulose fibers were crosslinked through the addition of glutaraldehyde, and Fe_3_O_4_ was loaded into the aerogel by dispersing Fe_3_O_4_ nanoparticles into the cellulose suspension through ultrasonication. The hydrophobization of magnetic cellulose aerogel was performed via chemical vapor deposition (CVD), using MTCS as the gaseous precursor. The color of HMCA changed from black to yellow–green after CVD, probably due to the formation of a small amount of iron(II) chloride tetrahydrate, which is the result of the reaction of Fe_3_O_4_ nanoparticles with HCl, a byproduct of MTCS hydrolysis. To optimize the concentration of Fe_3_O_4_, MCAs with different amounts of Fe_3_O_4_ nanoparticles were prepared, starting from 0.2 wt% and gradually increasing to 0.8 wt% using increments of 0.2 wt%. It was found that as the concentration of Fe_3_O_4_ increased, the adsorption capacity of MCA for pump oil decreased from 96.74 ± 0.89 to 36.89 ± 1.23 g/g. However, for the MCA of 0.2 wt% Fe_3_O_4_, the aerogel cannot be properly controlled by the external magnet; therefore, by comprehensively considering the magnetic properties and adsorption properties, aerogels with 0.4 wt% Fe_3_O_4_ were chosen as the optimal composition. Figure 2 shows the SEM images of CA (a,b), MCA (c,d), and HMCA (e,f). It can be seen that the pristine CA (Figure 2a,b) possesses a highly porous and staggered interconnected three-dimensional microstructure with the presence of sheet-like cellulose plates. The sheet-like cellulose plates composed of entangled cellulose fibrils form during the freezing process, in which the cellulose fibers are compressed by the growing ice crystals accumulating in front of them. After the addition of Fe_3_O_4_ nanoparticles, the color of the aerogel changed from white to black. Figure 2c shows that the staggered network structure with sheet-like plates does not change after the loading of Fe_3_O_4_ nanoparticles. The higher magnification image (Figure 2d) shows that Fe_3_O_4_ nanoparticles are uniformly dispersed on the surface of cellulose sheets. The strong hydrogen bonds formed between the cellulose structure and Fe_3_O_4_ nanoparticles are believed to be the main reason for this uniform distribution [26]. After CVD treatment with MTCS, a thin layer of polysiloxane particles forms (Figure 2e,f); therefore, the aerogel shows more particles on the surface. Although more particles are deposited on the cellulose sheets surface, the porous cellulose skeleton with cellulose sheets remains. The distribution of Fe_3_O_4_ and silane particles is characterized via EDX. As shown in Figure 1g, the Fe and Si elements are uniformly distributed on the surface of the cellulose sheet, which is in agreement with the SEM images. Table 1 shows the density of CA, MCA, and HMCA, respectively. All of the aerogels show low density and high porosity (>95%).

### 3.2. Characterization of CA, MCA, and HMCA

**Figure 3:**
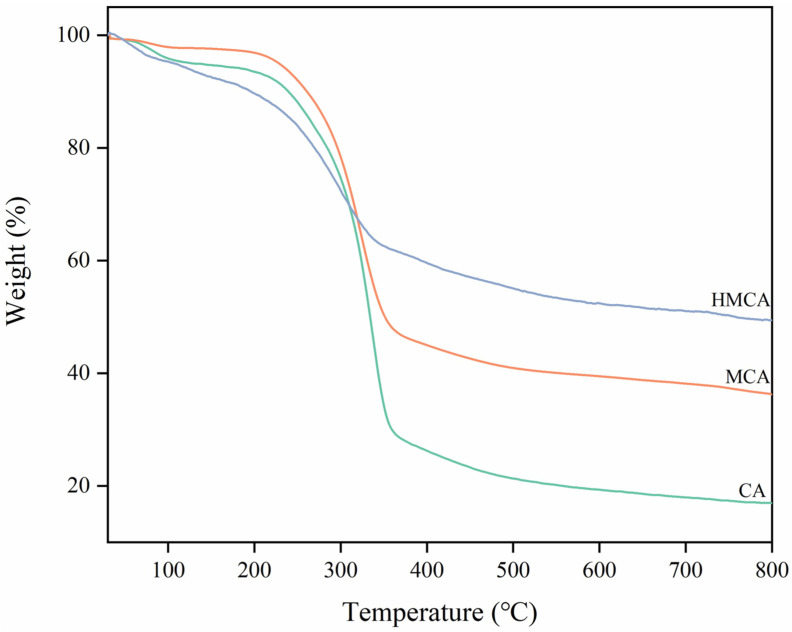
The thermogravimetric analyses of CA, MCA, and HMCA.

The thermogravimetric analyses of CA, MCA, and HMCA are shown in Figure 3. All three samples underwent a small weight decrease (˂10%) from the initial room temperature to 200 °C, which is ascribed to the evaporation of the absorbed water. The main weight loss that happened from 200 to 350 °C was mainly due to the thermal degradation of cellulose polysaccharide [27]. The residual weights for CA, MCA, and HMCA are 19%, 38% and 52%, respectively, indicating the weight percentages of the Fe_3_O_4_ nanoparticles and coated polysiloxane particles in the aerogels.

**Figure 4:**
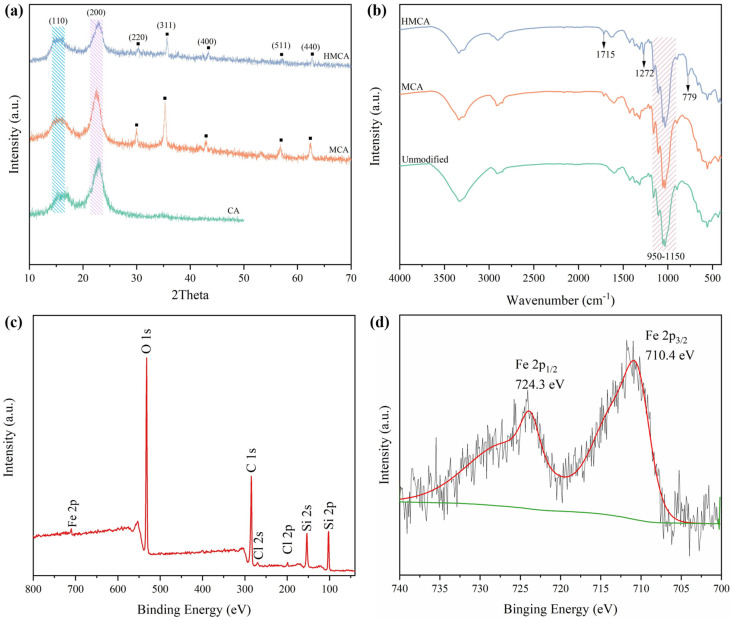
(**a**) XRD pattern of CA, MCA, and HMCA. (**b**) The FTIR spectra of unmodified cellulose aerogel, MCA, and HMCA. (**c**) Full-scan XPS spectra of HMCA. (**d**) XPS high-resolution Fe 2p scan of HMCA.

Figure 4a shows the XRD pattern of three aerogels. The unmodified CA exhibited two obvious peaks at 2θ = 16.2° and 22.5°, which are the characteristic peaks of cellulose, attributed to the (110) and (200) reflections [28]. After the addition of Fe_3_O_4_ nanoparticles, five new peaks at 30.1°, 35.6°, 43.2°, 57.0°, and 62.5°, corresponding to (220), (311), (400), (511), and (440) plane diffractions of Fe_3_O_4_, appeared on the MCA and HMCA XRD patterns [24]. Those characteristic Fe_3_O_4_ diffraction peaks confirmed the successful incorporation of Fe_3_O_4_ nanoparticles into the cellulose aerogel. No new peak or peak shift were seen from the silane modified aerogel, indicating that no new phase was introduced via this silanization process.

FTIR was applied to study the effects of the glutaraldehyde crosslinking of the cellulose aerogel. As shown in Figure 4b, all three samples exhibit the characteristic peaks of cellulose, including a wide band around 3700–2900 cm^−1^, with a broad peak around 3300–3350 cm^−1^, which is attributed to the O–H stretching; a peak at 2890 cm^−1^, which is assigned to the C–H stretching vibration; and a peak at 1029 cm^−1^, which is due to the C–O vibration of cellulose [29,30]. Compared to the unmodified cellulose aerogel, the FTIR spectrum of glutaraldehyde crosslinked aerogels (MCA) showed a higher peak intensity at 950–1150 cm^−1^, which is ascribed to C–O stretching. This is attributed to the successful crosslinking of glutaraldehyde, in which its C=O double bonds are open [31]. In addition, a new aldehyde peak appearing at 1715 cm^−1^ also confirmed the crosslinking of glutaraldehyde [32]. After hydrophobic modification via MTCS, two new peaks were seen on the FTIR spectra of HMCA at 779 cm^−1^ and 1272 cm^−1^. These two absorption peaks were attributed to the Si–C bond vibration and the –CH_3_ vibration of the polysiloxane particles, respectively. The appearance of those two new peaks indicates the successful MTCS modification of the aerogels. XPS analysis was carried out on the HMCA to study the chemical composition. The full-scan spectra (Figure 4c) shows seven peaks associated with Si 2p, Si 2s, Cl 2p, Cl 2s, C 1s, O 1s, and Fe 2p, which is in agreement with the EDX results. In the full-scan spectra, the peaks associated with chlorine are due to the formation of a small amount of FeCl_2_ during the MTCS CVD process, and the weak intensity indicates its content is very low. The high-resolution Fe 2p scan is shown in Figure 4d, and two peaks at 710.4 eV and 724.3 eV attributed to Fe 2p3/2 and Fe 2p1/2 spin orbit peaks of Fe_3_O_4_, respectively, are seen, which indicate that the Fe_3_O_4_ has been successfully incorporated into the aerogel [33].

### 3.3. Surface Wettability

**Figure 5:**
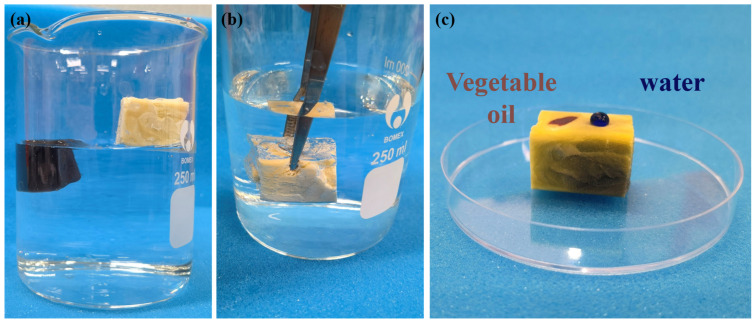
(**a**) MCA is sinking into the water, while HMCA is floating on the water surface. (**b**) Mirror-reflection phenomenon occurred when pushing HMCA under water. (**c**) Vegetable oil and water droplets on the surface of HMCA.

**Figure 6:**
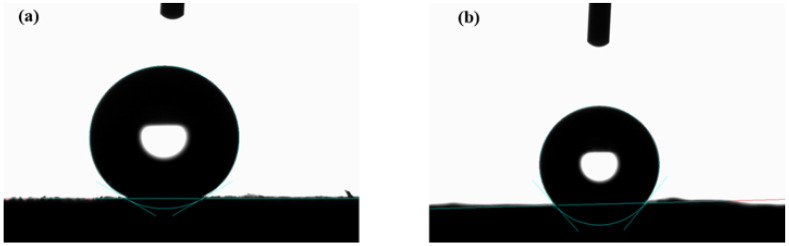
(**a**) Static water contact angle of HMCA. (**b**) Static water contact angle of MTCS-treated cellulose aerogel without addition of Fe_3_O_4_ nanoparticles.

The surface wettability of oil sorbent is crucial for achieving selective adsorption of oil. CA and MCA are hydrophilic due to the presence of a large number of hydroxyl groups. A straightforward chemical vapor deposition (CVD) process, using MTCS as a gaseous precursor, was carried out on the MCA to alter its surface wettability (Figure 1). Following CVD, the hydrophilic –OH groups were substituted with the polysiloxane particles, which contain the hydrophobic –CH_3_ terminal groups that rendered the aerogels superhydrophobic. The enhanced surface roughness resulting from the presence of polysiloxane particles further enhanced the HMCA’s hydrophobicity. As shown in Figure 5a, after MTCS modification, HMCA could float on the surface of the water, but CA and MCA sunk under the water surface. Figure 5b shows that when pushing HMCA under water, a mirror reflection was seen on the surface of the aerogel, which is due to the entrapped air between the aerogel surface and surrounding water. This mirror-reflection also refers to the Cassie–Baxter surface wetting model [34]. Figure 5c shows that water droplets (dyed with methylene blue) stood spherically on the surface of HMCA, while vegetable oils (dyed with Sudan red) were adsorbed and penetrated the aerogel. The wettability difference indicates the excellent oil adsorption selectivity of MTCS modified HMCA. The static water contact angle was measured to characterize the surface wettability of HMCA. As shown in Figure 6a, the water droplet stood spherically on the surface of HMCA with a contact angle of 150.3°, which illustrated its superhydrophobicity. The water contact of the MTCS-treated cellulose aerogel (without the addition of Fe_3_O_4_ nanoparticles) was also measured to study the effect of Fe_3_O_4_ nanoparticles on aerogel’s wettability. It was found that without the addition of Fe_3_O_4_ nanoparticles, the water contact angle of the MTCS-treated aerogel was 130° (Figure 6b), which was lower than the HMCA. This is attributed to the increased surface roughness of the Fe_3_O_4_ nanoparticles coating the aerogel surface.

### 3.4. Magnetic Property of HMCA

**Figure 7:**
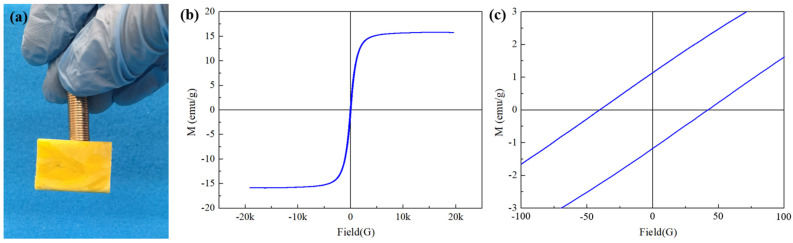
(**a**) Lifting HMCA with magnets. (**b**) Vibration sample magnetometer (VSM) measurement diagram of HMCA. (**c**).

Figure 7a shows that after the incorporation of Fe_3_O_4_ nanoparticles, the HMCA can be easily lifted by external magnets, which demonstrates its great response to an external magnetic field. Since the magnetic properties of HMCA are critical for its application, the magnetic properties of the aerogel were furthered investigated using VSM at room temperature, with a magnetic field strength of ±18,000 Oe, and the resultant hysteresis loops are shown in Figure 7b,c. From Figure 7b, the saturation magnetization (Ms) of HMCA is determined as 15.75 emu/g, and the remanence (Mr) and coercivity (Hc) are determined as 1.12 emu/g and 40.42 Oe, respectively, in Figure 7c. Due to the small values of Ms and Hc, HMCA is determined to have superparamagnetic behavior, and the high Ms value ensured the good magnetic response of HMCA to external magnetic fields.

### 3.5. Adsorption Capacity and Adsorption Kinetics of HMCA

**Figure 8:**
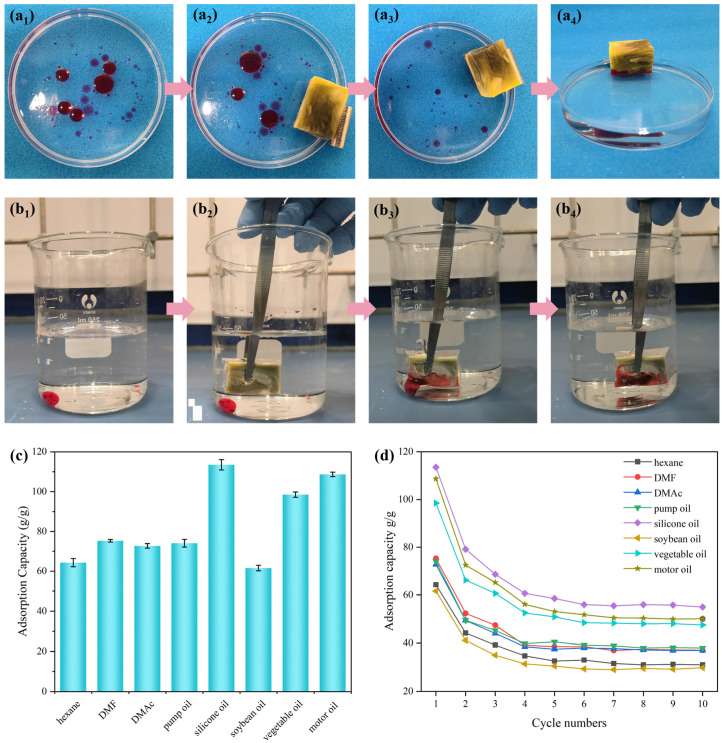
(**a**) Adsorption of vegetable oil at the surface of water. (**b**) Adsorption of chloroform below the water. (**c**) Adsorption capacity of HMCA for different types of oils and organic solvents. (**d**) Reusability of HMCA.

To investigate the performance of modified cellulose aerogels for oil absorption in water and assess their suitability for addressing marine oil pollution, laboratory tests were conducted to determine their oil-removal efficacy. As shown in Figure 8a,b, HMCA was placed in a beaker containing a mixture of motor oil (dyed with Sudan III) and water. Upon contact with the oil/water mixture, the aerogel began to soak up the red motor oil while floating on the water surface. This demonstrated the rapid absorption rate of HMCA and outstanding oil sorption selectivity. Figure 8(a_2_,a_3_) shows that HMCA can be controlled to move across the water surface using external magnets. Furthermore, after the aerogel reached its sorption saturation, it still floated on the water surface, and no oil flow back was observed from the aerogel, which indicates its excellent oil sorption capacity and oil retention. Figure 8b shows that when pushing HMCA under the water, it could also quickly absorb the chloroform (dyed with Sudan III). The adsorption process of non-polar oils and organic solvents can be divided into three steps: (1) the diffusion of oil molecules onto the aerogel surface, (2) the retention of organic liquid due to the capillary force, and (3) the accumulation of the adsorbed liquid into the pores of the aerogel [25,35]. Besides motor oil and chloroform, a variety of oils and organic solvents were selected to test the adsorption capacity of HMCA. The results are presented in Figure 8c. It was seen that HMCA showed a high adsorption capacity for all of the tested oils and organic solvents, and the maximum adsorption capacity for silicone oil was the highest at 113.49 g/g. This is mainly attributed to their superhydrophobicity and highly porous structure, which provides lots of space for 335 oils and organic solvents. Previous researchers have developed various oil sorbent materials with different oil adsorption capacities, including coir fiber-reinforced PVA aerogel (25–34 g/g) [36], silane-coated chitin sponge (29–58 g/g) [37], polydimethylsiloxane/carbonized bacterial cellulose sponge (3–9 g/g) [38], TiO2-coated nanocellulose aerogels (20–40 g/g) [39], polyurethane foam (62–65 g/g) [40], calcium stearate-coated kapok fibers (up to 59.69 g/g) [41], graphene aerogels (260–570 g/g) [42], etc. The magnetic cellulose aerogel prepared in this work has the potential for practical oil adsorption due to the (i) outstanding oil adsorption capacity of many types of oils and organic solvents, (ii) low-cost and feasible preparation process compared to the graphene-based oil sorbents, and (iii) environmental friendliness compared to the synthetic polymer-based oil sorbents, such as polyurethane and polydimethylsiloxane. Moreover, the oil adsorption capacity of this HMCA is also better than those of many of other cellulose-based aerogels. A comparison of the oil adsorption capacity of our HMCA with those of other cellulose-based aerogels is shown in Table 2.

The adsorption kinetics of HMCA was determined by dipping equal-sized samples of HMCA into pump oil for different times and measuring their weight differences. Pseudo-first-order (Equation (4)) and pseudo-second-order models (Equation (5)) were used to simulate the oil sorption kinetics of s-HNAs.

(4) $$\mathrm{Ln}\left(q_{m}-q_{t}\right)=lnq_{m}-k_{1}t$$

(5) $$\frac{t}{q_{t}}=\frac{1}{q_{m}}t+\frac{1}{k_{2}q_{m}^{2}}$$

where *q_m_* and *q_t_* (g/g) are the maximum sorption capacity and the sorption capacity at time *t*, respectively. *t* (s) is the time. *k*_1_ and *k*_2_ are the sorption rate constants [48].

**Figure 9:**
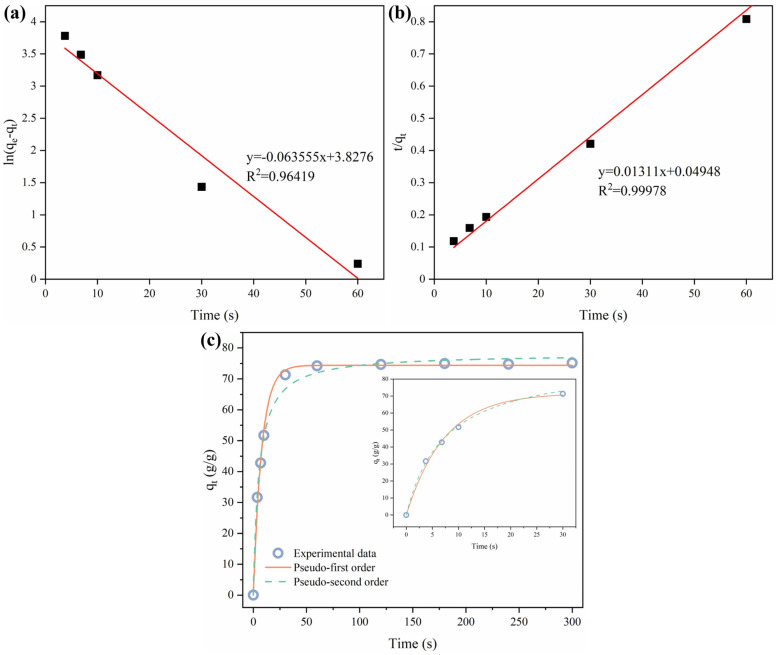
(**a**) Pseudo-first-order adsorption linear fitting of HMCA adsorb pump oil. (**b**) Pseudo-second-order adsorption linear fitting of HMCA adsorb pump oil. (**c**) Experimental data fitted with pseudo-first-order and pseudo-second-order models for the adsorption kinetics of HMCA-adsorbing pump oil.

For the adsorption of pump oil using HMCA at room temperature, Figure 9a,b shows the plots of *ln*(*q_m_* − *q_t_*) versus *t*, and *t*/*q_t_* versus *t*, corresponding to the pseudo-first-order and pseudo-second-order models, respectively. Figure 9c shows the experimental adsorption data and the fitted curves of the two adsorption models. The sorption constants are calculated based on the fitted curves, and the results are *k*_1_ = 0.06355 and *k*_2_ = 0.00351. The higher correlation factor (R^2^) value shown in Figure 9b indicates that the pseudo-second-order model is better for the prediction of the adsorption behavior of HMCA.

Furthermore, the reusability of HMCA is another critical factor for its practical application as an oil sorbent. The reusability of HMCA was characterized via the cyclic adsorption–squeezing method, and the corresponding adsorption capacity after each cycle is shown in Figure 8d. In a typical adsorption–squeezing test, the saturated aerogel was removed from the organic solvent and squeezed between two parallel glass slides, whereby >90% strain was applied. Then, the aerogel was immersed in the same solvent to reach adsorption saturation. The weight of the aerogel after each adsorption cycle was measured. The recyclability measurement results show that the adsorption capacity of HMCA has a big deterioration (loss about 38% maximum adsorption capacity) after the first squeeze and gradually reduces to a stable value after the fourth cycle. The reduction in the adsorption capacity is attributed to the permanent collapse of the porous structure caused by mechanical compression, which results in the loss of pore volumes for retaining the organic solvent. However, about 50% of the initial adsorption capacity of HMCA can be retained after five adsorption–squeezing cycles, which indicates its good reusability.

**Figure 10:**
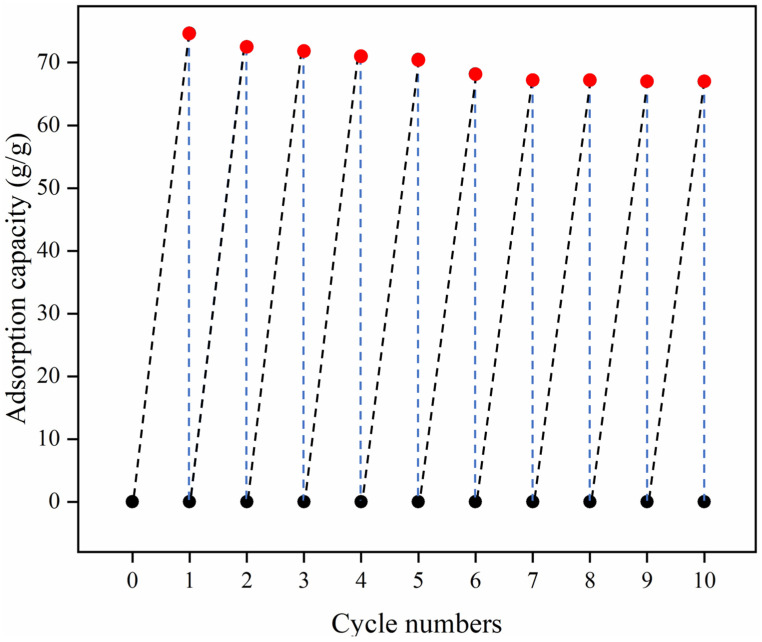
Reusability test of HMCA using adsorption–washing method.

Since the mechanical squeezing led to the permanent deformation of the internal structure of the aerogel, another rinsing method was applied to characterize the reusability of HMCA. In this method, the pump oil-saturated HMCAs were rinsed in ethanol to release the adsorbed oil. The sample was subsequently dried in a vacuum oven for 8 h at 70 °C, and the oil adsorption capacity was tested again. Almost no weight difference was observed between the ethanol rinsing, vacuum oven-dried HMCA, and HMCA before oil adsorption, indicating that this rinsing method can completely wash out the adsorbed oil. This adsorption–washing test was repeated 10 times, and the results are shown in Figure 10. It is found that the maximum adsorption capacity only decreased by about 10.5% after 10 adsorption–washing cycles, and the adsorption capacity tended to be stable.

## 4. Conclusions

A superhydrophobic magnetic cellulose-based aerogel was prepared in this work for the selective adsorption of oils and organic solvents from oily wastewater. The aerogel was prepared via an environmentally friendly and cost-effective method. It was found that the incorporation of Fe_3_O_4_ nanoparticles not only endowed the aerogel with superparamagnetic properties, but also contributed to better hydrophobicity. The water contact angle was surprisingly increased for the aerogel with Fe_3_O_4_ nanoparticles, and this was believed to be the result of higher surface roughness caused by the incorporation of Fe_3_O_4_. VSM testing shows that the HMCA is superparamagnetic, with a good response to external magnetic fields. The highly porous structure and hydrophobic modification were the main reasons for the outstanding oil adsorption capacity of HMCA (up to 113.49 g/g for silicon oil). The recycling test showed that the prepared HMCA can preserve about 50% of its maximum adsorption capacity after 10 adsorption–squeezing cycles. Moreover, the aerogel only showed an about 10.5% decline in the adsorption capacity if the washing method was applied to release the adsorbed oils, which indicates its great reusability. Combining these outstanding properties, this material has great potential to be used as an environmentally friendly and economical adsorbent for the treatment of oily wastewater.

## Figures and Tables

**Table 1:** Density and porosity of CA, MCA, and HMCA.

	CA	MCA	HMCA
Density (mg/cm^3^)	9.47	14.63	18.25
Porosity (%)	99.38	99.04	98.80

**Table 2:** Comparison of the oil adsorption capacity of our work with other cellulose-based aerogels.

Material	Adsorption Capacity (g/g)	Ref.
Cellulosic aerogel from water hyacinth	40.40–43.3	[43]
TiO_2_-coated nanocellulose aerogels	20–40	[39]
Cellulose/tannic acid/castor oil aerogels	53.2–113.8	[44]
Nanoalumina-loaded nanocellulose aerogel	64.83–117.64	[45]
Hybrid silica–cellulose aerogel	24.8	[46]
Cellulose nanofibers/alginate aerogels	88.91	[47]
HMCA	61.56–113.49	This work

## Data Availability

The data presented in this study are available on request from the corresponding author.

## Supplementary Materials

The following supporting information can be downloaded at: https://www.mdpi.com/article/10.3390/polym15193941/s1, Figure S1. SEM image of the cellulose nanofibers isolated from harakeke.
