# Supplementary material for: Superhydrophobic, Magnetic Aerogels Based on Nanocellulose Fibers Derived from Harakeke for Oily Wastewater Remediation
Source: Polymers (Basel). 2023 Sep 29;15(19):3941. doi: 10.3390/polym15193941 (PMC10575082; doi:10.3390/polym15193941)
Supplement: Supplementary file 1 [file polymers-15-03941-s001.zip › polymers-2626202-supplementary.pdf]

Superhydrophobic, magnetic aerogels based on nanocellulose fibers derived from harakeke for oily wastewater remediation

Yitong Zhai[a], Xiaowen Yuan[a*]

[a] Future Fibres Laboratory, School of Engineering, Computer and Mathematical Sciences, Auckland University of Technology, Auckland, New Zealand.

[*]Corresponding author: Xiaowen Yuan

E-mail: xiaowen.yuan@aut.ac.nz

Phone: +64 9 921 9999 ext 7320

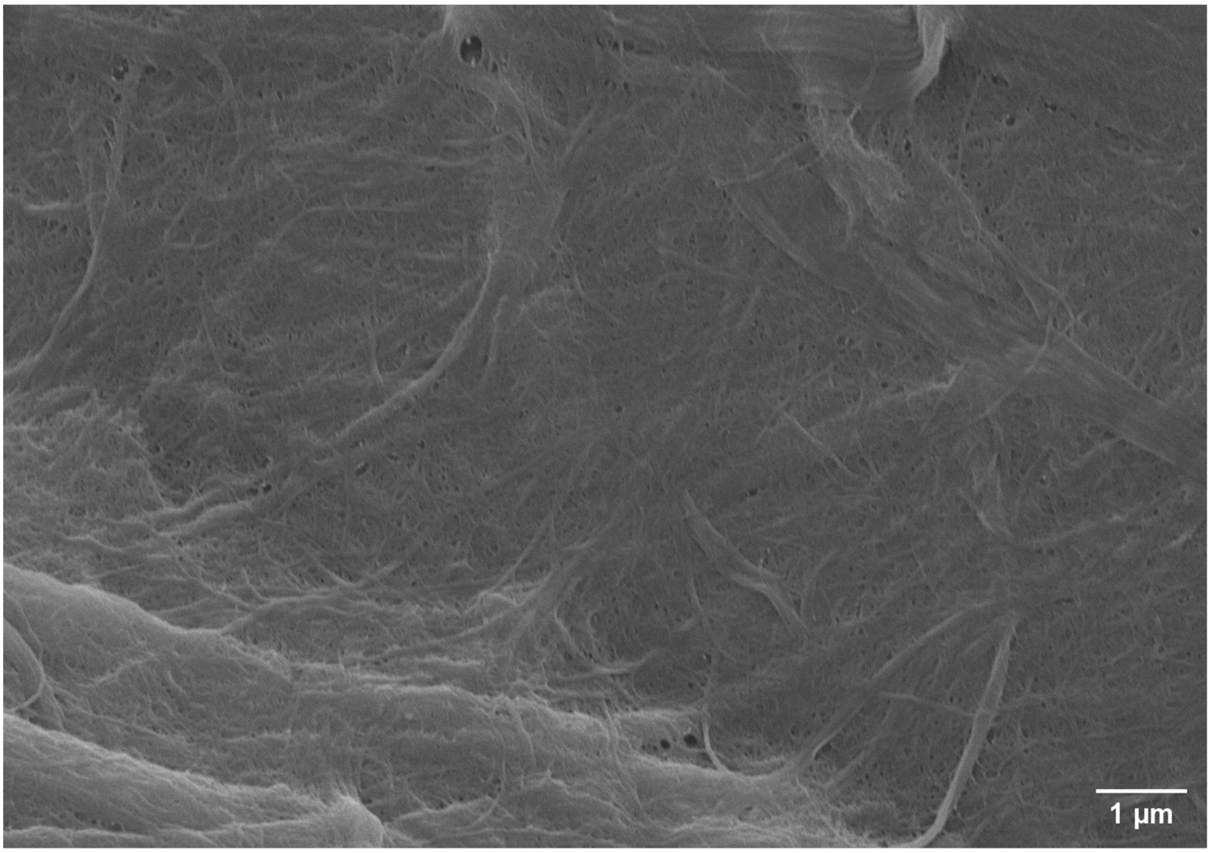

Figure S1. SEM image of the cellulose nanofibers isolated from harakeke.
